# Publisher Correction: Cyrius: accurate *CYP2D6* genotyping using whole-genome sequencing data

**DOI:** 10.1038/s41397-022-00287-3

**Published:** 2022-09-28

**Authors:** Xiao Chen, Fei Shen, Nina Gonzaludo, Alka Malhotra, Cande Rogert, Ryan J. Taft, David R. Bentley, Michael A. Eberle

**Affiliations:** 1 Illumina Inc., 5200 Illumina Way, San Diego, CA USA; 2 Illumina Cambridge Ltd., Illumina Centre, 19 Granta Park, Great Abington, Cambridge, UK

**Keywords:** Predictive medicine, Pharmacogenomics

Correction to: *The Pharmacogenomics Journal* 10.1038/s41397-020-00205-5, published online 18 January 2021

The copyright holder for this article was incorrectly given as ‘The Author(s), under exclusive licence to Springer Nature Limited 2021’ but should have been ‘The Author(s) 2021’.

Further the licence information was missing from this article and has now been added.

The original article has been corrected.

